# Clinical applications and challenges in the field of extracellular vesicles

**DOI:** 10.1515/medgen-2023-2062

**Published:** 2023-12-05

**Authors:** Rienk Nieuwland, Agustin Enciso-Martinez, Jillian W.P. Bracht

**Affiliations:** Amsterdam UMC, location University of Amsterdam Amsterdam Vesicle Center, Laboratory of Experimental Clinical Chemistry, Department of Clinical Chemistry Amsterdam The Netherlands; Amsterdam UMC location University of Amsterdam, Amsterdam Vesicle Center, Laboratory of Experimental Clinical Chemistry, Department of Clinical Chemistry Amsterdam The Netherlands; Amsterdam UMC, location AMC Amsterdam Vesicle Center, Laboratory of Experimental Clinical Chemistry, Department of Clinical Chemistry Amsterdam The Netherlands

## Abstract

Body fluids contain cell-derived particles called extracellular vesicles (EVs). EVs are released by cells and are present in all body fluids (i. e. liquid biopsies). EVs contribute to physiology and pathology and offer a plethora of potential clinical applications, ranging from biomarkers to therapeutic applications. In this manuscript we provide an overview of this new and rapidly growing research field, along with its challenges and opportunities.

## Introduction

Extracellular vesicles (EVs) is an umbrella term introduced by the International Society for Extracellular Vesicles (ISEV; www.isev.org). The term “extracellular vesicles” encompasses all membrane-delimited particles that are released from cells into their environment. These particles or vesicles are highly heterogeneous, and differ in size, the underlying mechanism of release, cellular origin, biochemical composition, physical properties, and function.^1^ EVs are present in all fluids contacting or containing cells, including body fluids (e. g. liquid biopsies).

**Figure 1.**
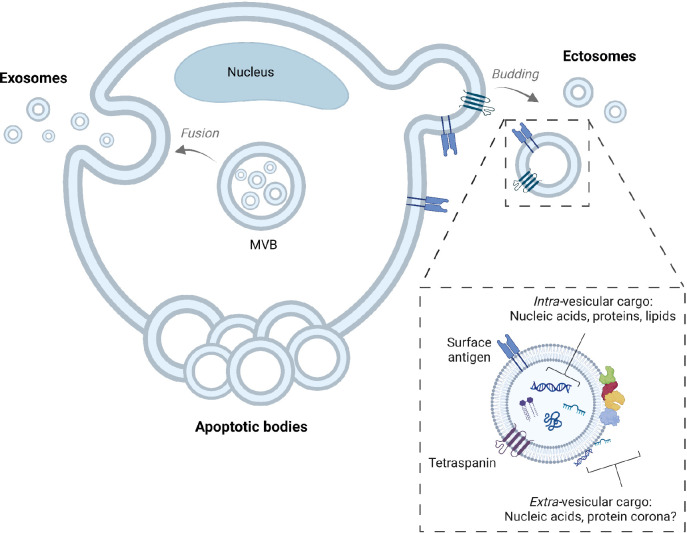
Generation of extracellular vesicles Extracellular vesicles (EVs) include exosomes, ectosomes (also known as microvesicles or microparticles), and apoptotic bodies. Exosomes are stored as intraluminal vesicles in multivesicular bodies (MVBs). When the MVB membrane fuses with the plasma membrane, the intraluminal vesicles are released into the extracellular environment. When present outside the cell, these vesicles are called exosomes. Ectosomes are formed due to direct budding from the plasma membrane. When cells are undergoing programmed cell death or apoptosis, cells can fragment into apoptotic bodies. Exosomes, ectosomes and apoptotic bodies overlap in size, density and biochemical composition, which makes it impossible to distinguish these subcategories. Therefore, we recommend using the umbrella term “extracellular vesicles”. An example of the biochemical composition of EVs is also shown, including tetraspanins and a transmembrane protein with a unique surface antigen that can be used to determine the cellular origin of a single EV. Finally, also the EV-associated cargo has been depicted, showing that EVs may carry nucleic acids, proteins and lipids in their lumen and/or on their surface membrane. EVs: extracellular vesicles; MVB: multivesicular body. The protein corona is not shown. Figure created with BioRender.com.

Regarding the underlying release mechanisms, cells can release EVs directly by budding from the plasma membrane, or by secreting “ready-to-go” EVs which are stored in intracellular granules, as shown in Figure 1. EVs released from the plasma membrane are called microparticles, microvesicles or ectosomes, whereas EVs released from the intracellular storage granules, known as multivesicular bodies or multivesicular endosomes, are called exosomes. Currently there is still no reliable way to properly distinguish between the different EV types.

Regarding their cellular origin, EVs may express cell-type specific markers originating from their parental cells. To determine the cellular origin of single EVs, the presence of cell-type specific markers can be detected by methods such as flow cytometry.^2^ For example, to identify EVs in human blood plasma, EVs can be stained using (fluorochrome-labelled) antibodies directed against cluster of differentiation (CD) markers, usually transmembrane proteins, such as CD235a for erythrocyte-derived EVs, CD61 for platelet-derived EVs, and CD45 for leukocyte-derived EVs.^3^ The biochemical composition of EVs will also depend on the “status” of the cell releasing the EVs. For example, activated but not resting human platelets expose an adhesion receptor called P-selectin (CD62p), and this adhesion receptor can only be present on EVs released from activated platelets.^3^

The majority of EVs range in diameter from 30 to 1000 nm.^4^ Cells can also release larger vesicles such as apoptotic bodies or large oncosomes, which both may also have value as biomarkers. In this review we will focus on the aforementioned ectosomes and exosomes, which we will refer to as “extracellular vesicles” (EVs) since their subcellular origin, and hence the type of EV cannot be reliably established. We thus recommend using the term “extracellular vesicles” (EVs) for all membrane-delimited particles that are released from cells into their environment.

## Presence of extracellular vesicles

All fluids that contain, or are in contact with cells contain EVs. There are reports that *in vitro* conditioned cell culture media, ocean water, body fluids and consumables such as beer contain EVs. These EVs can be of both eukaryotic and prokaryotic origin, and their contribution to for example interkingdom communication is a topic of growing interest.

## Biochemical composition of extracellular vesicles

EVs are membrane-delimited particles. This means that most EVs are spherical particles surrounded by a phospholipid bilayer membrane. Similar to a cell containing cytosol, an EV contains intra-vesicular fluid. EVs are mainly composed of lipids and proteins, and EVs are thought to be carriers of DNA and RNA (Figure 1).^5^

The particle size distribution of EVs in human plasma reveals that there are no separate populations of large- and small EVs, but that the size distribution is tilted towards high concentrations of small EVs and relatively low concentrations of large EVs.^2,4,6^ It is important to keep in mind that because most EVs are small, having a diameter of less than 200 nm, the total population of EVs has a surprisingly large surface to volume ratio. For example, for human plasma EVs, assuming a concentration of 10^10^ EVs/mL and a size distribution as reported earlier,^4^ the total surface area per mL is 1 600 mm^2^ compared to a total volume of only 50 nL. Consequently, the high concentrations of small EVs provide most of the surface, whereas the bulk of the volume is provided by the relatively sparse large EVs. Especially this large surface area is intriguing and recently several research groups independently described the presence of a protein corona on the surface of EVs, meaning that carefully isolated EVs from plasma are still coated or covered with a particular subset of plasma proteins.^7,8^ To which extent the intra-vesicular lumen, the membrane surface and/or the corona of EVs are involved in containing, binding and/or stabilizing and transporting DNA and/or RNA, is currently under investigation.

## Isolation of extracellular vesicles

EVs are not only heterogenous in size and in biochemical composition, but they can also be outnumbered by non-EV particles in body fluids such as blood plasma and milk. Therefore, to gain insight into the biochemical composition of EVs, separation of EVs from the other non-EV components is essential prior to EV analysis^9^.

As a first step after collection of a body fluid or liquid biopsy, the cells are separated from the EV-containing fluid by centrifugation. Removal of cells is important because cells will fragment during a freeze-thaw cycle, and cell fragments will be indistinguishable from EVs.^10^ Furthermore, cells may also be difficult to separate from EVs in further EV isolation steps.^11^ The EV-containing body fluids or liquid biopsies are the starting material for EV research, either directly, or after storage. Storage in biobanks is common practice, and there is increasing awareness that the consistency and quality of the prepared samples (e. g. with regard to the efficacy of cell depletion) are important parameters to be quantified and reported, since they are likely to help better understand the results of downstream analyses.

After removal of cells, the EVs can be isolated. Current isolation or separation methods are based on (differences in) size, density, charge and biochemical composition. Examples of commonly used separation methods are (i) size-exclusion chromatography (SEC), which is a size-based separation method, (ii) density gradient centrifugation, a density-based separation method, (iii) antibody-coated capture beads, which is a separation method based on differences in the biochemical composition, and (iv) ultracentrifugation, which is a size and density-based separation method. However, isolation of EVs is neither easy nor straightforward, and the choice of separation method may strongly affect the yield and purity of EVs.^12^ For example, after centrifugation of human blood to remove cells, the collected plasma will contain mainly non-EV particles, such as lipoproteins, as well as a low concentration of remaining platelets.^10,13^ Some lipoproteins, such as high density lipoproteins (HDL) can outnumber the concentration of EVs by a million-fold.^14^ Since platelets and lipoproteins can overlap in size and density with EVs, it is challenging to separate such non-EV particles from EVs using currently available separation methods. Since optimal separation of EVs from confounders is difficult, it is important to quantify relevant confounders in the “isolation fractions of EVs”. In the example of human plasma, plasma contains multiple carriers of miRNA, including platelets, soluble proteins, lipoproteins and EVs. Thus, in order to be able to attribute the presence of miRNAs to plasma EVs, the presence of miRNA carriers other than EVs should be quantified and reported to prevent over-interpretation of results ^10^.

There is growing awareness that isolation of EVs is difficult. Recently, there is a tendency to combine separation methods based on different isolation principles. For example, after cell removal by centrifugation, as a next step EVs can be isolated using a size-based separation method such as SEC. After collection of the EV-enriched SEC fractions, these SEC fractions are subjected to a density-based fractionation, to separate EVs from (non-EV) particles with a different density.^15^ However, one has to take into account that every isolation step will decrease the total yield of EVs. Finally, for some assays, isolation of EVs is not required. For example, body fluids as normal human saliva and milk contain EVs exposing tissue factor (TF), a transmembrane receptor for coagulation factor VII. EV-exposed TF triggers coagulation, and this coagulant activity (exclusively associated with EVs) is so strong, that addition of these fluids to human plasma will directly trigger coagulation and clotting of plasma.^16^ In other words, the biological activity associated with these EVs does not require isolation because EVs are the only carriers of this biological activity.

## Detection of extracelluar vesicles

In this section, we will focus only on detection methods that detect intact EVs. Two early methods are still commonly used to detect EVs, i. e. Nanoparticle Tracking Analysis (NTA) and Tunable Resistive Pulse Sensing (TRPS). Both methods detect single particles and not exclusively EVs, thereby hampering their applicability for detection of EVs in body fluids and liquid biopsies. Since then, many more methods have been developed. In general, these methods can be classified as methods detecting EVs either in suspension or on a surface, and, in addition, as methods detecting single EVs or multiple EVs simultaneously. For example, flow cytometry detects single particles (including EVs) directly in suspension.^2^ Recently, ISEV started a new online course explaining the principles of various EV detection and isolation methods, and we recommend this course for more information (www.isev.org/education).

## Physiological and pathological functions of extracellular vesicles

So why do cells release EVs, what are their functions? Initially, EVs were discovered as subcellular particles present in human plasma that promote coagulation^17^, and as particles that remove transferrin, a redundant transmembrane receptor,from maturing red blood cells.^18^ What these initial discoveries have in common, is that the membrane surface of EVs plays a key role. As explained, EVs have a large surface to volume ratio, and both coagulation and removal of a redundant transmembrane receptor require or benefit from a large surface. Also, what both processes seem to have in common, is that they contribute to homeostasis or protection. Coagulation contributes to haemostasis, the process that stops bleeding and restores the body’s integrity, the “milieu-interieur”, so that the chance that microorganisms enter the body is reduced. In the example of the transferrin receptor, the cell is protected from accumulation of a redundant receptor. There is increasing evidence that EVs play a role in protection, for example by promoting coagulation and inflammation, but there is also evidence that EVs may be carriers of signalling molecules or genetic information to target cells.^19,20^ As explained, isolation of exclusively EVs, even from conditioned culture medium, is not easy, and there are still believers and non-believers regarding the relevance of the role that EVs may play in intercellular communication, for example by transferring RNA from one cell to the other via EVs. Less than 1 % of total miRNA present in human plasma is associated with EVs^21^, and since it is not possible to really purify EVs to such an extent that no confounders are present, it is still a matter of debate whether such EVs contain or do not contain miRNAs.^22^ Also, the fact that simply the intra-vesicular space may be insufficient to store a single miRNA, makes one wonder whether miRNA and other cargo are stored on the surface or in the lumen of EVs. Recent improvements in detection and isolation methods, combined with the recent discovery of a protein corona^23^, and the fact that DNA seems to be present mainly on the surface of EVs, may tip the balance.

Many more functions of EVs have been described, ranging from complement activation, a protection mechanism benefiting from a large membrane surface, to promoting tumour growth and metastasis.^24,25^ Also, EVs have been shown to act as decoys to distract bacteriophages, which again is a form of protection.^26^ To which extent all functions currently reported will hold over time, remains to be determined, but the fact that EVs are really there and that complex intercellular machinery as ESCRT complexes are involved in cargo sorting, suggests that EVs are not merely innocent bystanders, but may really have multiple active roles in physiology and pathology.

## Overcoming the challenges in standardization of extracellular vesicle research

As explained, EVs are present in all body fluids, small in size and heterogeneous in composition, and detection, isolation and characterization methods are complicated. Consequently, extracting relevant information from EVs is neither easy nor straightforward. There is a growing awareness within the “EV community” that there is a need to improve standardization and reproducibility.^27^

There are multiple reasons why standardization and reproducibility of EV research should be improved. Firstly, in the United States alone annually USD 28,000,000,000 is wasted on irreproducible preclinical research.^28^ Secondly, biomarker development requires robust standardization and reproducibility. For example, the measured concentration of blood cells is approximately the same in all hospital laboratories. To measure the same concentration on instruments from different companies and brands, the instruments must be calibrated. Calibration requires a dedicated infrastructure of reference materials, test samples to validate reference materials, etc.^2^ Without a calibration infrastructure, the measurement results will be incomparable. This can be illustrated by the reported concentration of EVs in normal human plasma, which has been reported to range between 10^4^ and 10^12^ per mL during the last decade. Thus, the lack of calibration infrastructure hampers the comparability of EV measurement results. Thirdly, currently there are no established protocols for collection and handling of EV-containing (body) fluids, and there are no methods capable of isolating or detecting all or exclusively EVs.^29^ Fourthly, long-term credibility in a research field is lost when research findings are incomparable. This leads to a less attractive environment for funding agencies and industry to invest causing major problems for the EV community.

Thus, there is a growing need for reproducible research on EVs. Right now, we are at a stage that still most if not all studies on EVs are single-centre studies. We are entering a next phase, in which method development, calibration, transparent reporting (guidelines), interlaboratory comparison studies and standardization of pre-analytical procedures are evolving. A good example is flow cytometry. For about 25 years, EVs have been studied using flow cytometry, a method in which information of single particles, present in a suspension, is collected. This information can be used to determine the presence of epitopes present on the outside of the EVs, e. g. to establish their cellular origin, as well as their size (diameter), refractive index, and concentration.^2^ Because flow cytometers differ in sensitivity of detectors and in the volume they can analyse per unit of time, and because the particle size distribution of EVs is not even but tilted in the favour of smaller EVs being more frequently present than larger ones, even relatively small differences in sensitivity can already produce data that are incomparable between instruments.^6,27^ Together with European metrology institutes, a calibration infrastructure has been developed to calibrate flow cytometers used to detect EVs. Moreover, a framework for transparent reporting of EV flow cytometry measurement results has been introduced, which is now supported not only by ISEV, but also by the International Society on Thrombosis and Haemostasis (ISTH) and the International Society on Advancement of Cytometry (ISAC).^30^ Interlaboratory comparison studies have been organized to compare concentration measurements of EVs,^27^ and in the most recent global interlaboratory study, for the very first time all aspects of flow cytometry (flow rate, light scatter and fluorescence) have been calibrated (manuscript in preparation).

**Figure 2.**
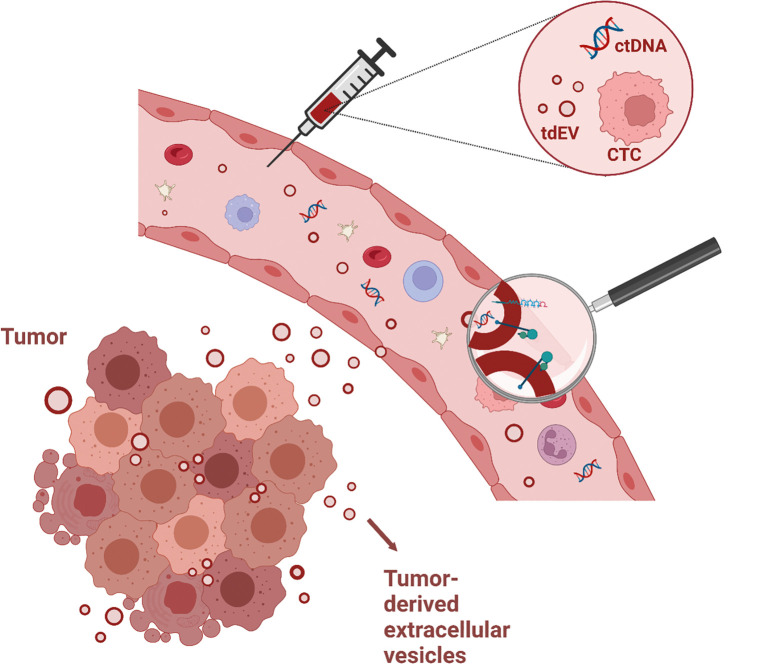
Biomarker potential of extracellular vesicles This figure illustrates potential biomarkers in liquid biopsies, namely circulating tumor cells (CTCs), circulating tumor-DNA (ctDNA), and circulating tumor-derived extracellular vesicles (tdEVs). A tumor (shown left) releases particles, which enter the blood. When a liquid biopsy, in this case blood, is collected, it contains traces originating from the tumor. Among these traces, tdEVs can contain tumor-specific markers (magnifying glass), including proteins, glycans, DNA and RNA. Hence, the detection and characterization of such tdEVs can provide clinically relevant information for diagnosis and disease monitoring.

More in general, ISEV established a Rigor and Standardization Subcommittee in 2019, which is now composed of task forces, working groups and special interest groups (www.isev.org/rigor-standardization). Especially the task forces focus on improving reproducibility of EV research in a particular area of interest, which ranges from a particular body fluid to regulatory affairs and therapeutics. Recently, several overviews have summarized what is going on regarding improving reproducibility of EV research.^31,32^

## Clinical applications of extracellular vesicle-derived biomarkers

Due to the role of EVs in intercellular communication, EVs and their cargo (e. g. proteins, lipids, DNA and RNA) may be valuable sources of biomarkers. For instance a liquid biopsy taken from a cancer patient includes tumour-derived EVs that present biomarkers which can be used for diagnostic, prognostic, predictive and disease monitoring purposes (see Figure 2 for an example of the biomarker potential of EVs). A diagnostic biomarker is measured to confirm the presence of a disease, while a prognostic biomarker can be used to identify disease recurrence or progression. A predictive biomarker can help to determine if a patient will benefit from a certain intervention. Finally, a monitoring biomarker is used to assess the status or extent of a disease and to evaluate the response to an intervention.

At present, EV-based biomarkers are being discovered from different EV cargoes. For example, testing for specific mutations for clinical decision making can be performed on EV-associated genomic DNA,^19,33^ whereas EV-associated (mi)RNAs have been used to predict treatment response.^20,34,35^ Biomarkers can also be derived from the lipid composition of EVs during pathogenesis.^36^ Finally, proteins spanning the EV membrane determine which cell types are more likely to take up the EVs (tropism), which is associated with the location of tumor metastasis.^24^

Although a huge effort of research into EV-based biomarkers is ongoing, in practice, the path from bench to clinical relevant EV biomarker development is not easily crossed.^37^ Although in 2021 more than 1 000 articles were published on EV-based biomarkers, only four EV-based biomarker assays have reached the phase of clinical validation.^38^ These four assays are ExoDX™ Prostate (IntelliScore) test, also known as “EPI test”^35,39^, the miR Sentinel™ Prostate Cancer Test or miR Sentinel™ PCC4 Test^20,40^, the miR Sentinel BCa™ Test (clinical trial NCT04155359), and the ClarityDx™ Prostate® test.^41,42^

Clearly, the attrition rate in the development of EV-based biomarkers is high. Several challenges underlying this high attrition rate have been mentioned in the previous sections, including the lack of standardized and robust methodologies. Besides the lack of standardization of collection, handling and isolation of EVs in general, the different methodologies used to isolate EV-associated *cargo* (e. g. RNA, DNA, lipids or proteins) introduces yet another variable.^10^ Since we also often don’t know yet whether the cargo of interest is located inside EVs, is present on the surface of EVs, or is located both inside and on the EV surface (also known as the “topology”), further hampers standardization of the used methodologies to isolate EV-associated cargo.

## Extracellular vesicle-based therapeutics

Since EVs can transport functional molecules, EVs can be used to carry and deliver therapeutic molecules *in vivo*, including proteins, RNA and chemotherapeutics.^43–45^ In general, using EVs as a delivery vehicle is safe, since autologous EVs are non-immunogenic and biodegradable. However, the large-scale manufacturing requirements, the pharmacokinetics of EVs *in vivo* (e. g. half-life), the systemic distribution of therapeutic EVs, and the loading and release of the functional molecular cargo are important factors that require optimization to make sure therapeutic molecules reach their target of interest.^44,46^

In a literature search performed for a manuscript on EVs derived from conditioned culture media (under review), 1 289 registered clinical trials involving EVs (trials.gov) were found when using the search terms “exosome” OR “microvesicle” OR “EV” (Jan 17^th^, 2023), of which most studies involve mesenchymal stem cell-derived EVs. Clearly, there is a strong and growing interest in using EVs not only for biomarker applications but also for therapeutic applications.

Taken together, although method development is ongoing, real clinical applications of EVs are gaining great interest. As such, EVs, initially considered as an artefact^47^, are turning into a real new entity with a strong clinical potential. It is not easy to provide a concise overview of a new and rapidly growing field of biomedical research, but we do hope that this overview may help to understand where the field really is, and what we may expect from EV research in the near future.
